# Fenofibrate Improves Insulin Resistance and Hepatic Steatosis and Regulates the Let-7/SERCA2b Axis in High-Fat Diet-Induced Non-Alcoholic Fatty Liver Disease Mice

**DOI:** 10.3389/fphar.2021.770652

**Published:** 2022-01-19

**Authors:** Dan Zhang, Shanzhuang Niu, Yicheng Ma, Hang Chen, Yu Wen, Mingke Li, Bo Zhou, Yi Deng, Chunjing Shi, Guangyu Pu, Meng Yang, Xianmei Wang, Chenggang Zou, Yuanli Chen, Lanqing Ma

**Affiliations:** ^1^ The First Affiliated Hospital, Yunnan Institute of Digestive Disease, Yunnan Clinical Research Center for Digestive Diseases, Kunming Medical University, Kunming, China; ^2^ State Key Laboratory for Conservation and Utilization of Bio-Resources in Yunnan, School of Life Sciences, Yunnan University, Kunming, China; ^3^ Faculty of Basic Medicine, Kunming Medical University, Kunming, China

**Keywords:** non-alcoholic fatty liver disease, fenofibrate, endoplasmic reticulum stress, let-7, microRNA, sarco/endoplasmic reticulum calcium ATPase

## Abstract

Fenofibrate is widely used in clinical therapy to effectively ameliorate the development of non-alcoholic fatty liver disease (NAFLD); however, its specific molecular mechanism of action remains largely unknown. MicroRNAs (miRNAs) are key mediators in regulating endoplasmic reticulum (ER) stress during NAFLD, and the deregulation of miRNAs has been demonstrated in NAFLD pathophysiology. The present study aimed to identify whether fenofibrate could influence miRNA expression in NAFLD and investigate the specific mechanism of action of fenofibrate in lipid metabolism disorder-associated diseases. We found that fenofibrate alleviated ER stress and increased the levels of SERCA2b, which serves as a regulator of ER stress. Additionally, the levels of let-7 miRNA were regulated by fenofibrate; let-7 was found to target the 3′ untranslated region of SERCA2b. The present data suggest that the protective effects of fenofibrate against insulin resistance and its suppressive activity against excessive hepatic lipid accumulation may be related to the alteration of the let-7/SERCA2b axis and alleviation of ER stress.

## Introduction

Non-alcoholic fatty liver disease (NAFLD) is typically characterized by excessive accumulation of abnormal amounts of lipids in hepatocytes (Antonucci et al., 2017; Quesada-Vázquez et al., 2020). Excessive hepatic lipid accumulation is related to an increased risk of chronic disease (Morán-Salvador et al., 2011; Adams et al., 2017; Chen et al., 2019). Therefore, understanding the mechanism underlying NAFLD development could help devise therapeutic strategies against this disease.

Accumulating evidence has revealed that hepatic endoplasmic reticulum (ER) stress critically promotes the development of NAFLD (Puri et al., 2008). The ER is present in the cytoplasm of eukaryotic cells, and it performs multiple essential functions, such as calcium storage and protein and lipid synthesis (Amen et al., 2019). ER stress occurs when ER function is disturbed by misfolded protein accumulation or depleted ER calcium levels. Hypoxia, energy disturbance, oxidative stress and other pathological conditions can trigger ER stress (Choy et al., 2018; Amen et al., 2019). Unfolded protein response (UPR) is a signal transduction network activated by ER stress (Yadav et al., 2014), which is initiated by some ER stress sensor proteins (Minamino et al., 2010; Gardner et al., 2013). For instance, activated inositol-requiring enzyme 1α (IRE1α) specifically cleaves X-box binding protein 1 (*XBP1*) mRNA, which is necessary for the translation of transcriptionally active XBP1 (Minamino et al., 2010). During ER stress, activating transcription factor 6 (ATF6) is activated following release from the ER chaperone, Grp78/BiP (BiP), which mediates the expression of UPR target genes, including *XBP1*. Although previous studies have shown the possible role of ER stress in NAFLD (Kim et al., 2018), the specific mechanism remains largely unknown. Sarco/endoplasmic reticulum calcium ATPase (SERCA) is an ER membrane-bound calcium pump (Zhang et al., 2020). Among the SERCA family members, SERCA2b plays an important role in cellular calcium homeostasis (Ushioda et al., 2016). The overexpression of SERCA2b improves ER stress and ameliorates NAFLD phenotypes in mice with obesity (Park et al., 2010; Fu et al., 2011; Chemaly et al., 2018). These results confirm that SERCA2b regulates ER stress during NAFLD.

MicroRNAs (miRNAs) are short non-coding RNAs that target the 3′ untranslated region (UTR) of an mRNA and regulate gene expression (Bartel, 2009). Previous studies have shown that aberrant miRNA expression contributes to metabolic disorders associated with NAFLD by altering key signaling elements (Trajkovski et al., 2011; Ng et al., 2014; Wu et al., 2017; Dai et al., 2019; Gjorgjieva et al., 2019). For instance, the overexpression of let-7 miRNA disrupts the glucose balance in mice and knockdown of let-7 attenuates hepatic lipid accumulation in diet-induced obese (DIO) mice (Frost and Olson, 2011). The action of let-7 occurs via the inhibition of the insulin-PI3K pathway by targeting the 3′ UTR of pathway components (Frost and Olson, 2011; Zhu et al., 2011). Our recent study verified that miR-30b regulates SERCA2b by targeting the 3′ UTR of *SERCA2b* mRNA, thus inducing ER stress and insulin resistance in DIO rats (Dai et al., 2019). Therefore, miRNAs represent potential novel therapeutic targets for NAFLD. Fenofibrate, a peroxisome proliferator-activated receptor α (PPAR-α) agonist, is a prescription medication used to lower cholesterol and triglyceride (TG) levels (Vardanyan and Hruby, 2016). It has been shown that fenofibrate improves fibrosis, inflammation, and hepatic lipid homeostasis by activating PPAR-α (Chanda et al., 2009), but its precise mechanism of action is not completely understood. The primary objective of the present study was to investigate whether fenofibrate improves ER stress and regulates the expression of let-7 in high-fat diet (HFD)-induced NAFLD mice and determine the potential target of let-7.

## Materials and Methods

### Animals

Male C57BL6/J mice (6 weeks old) were purchased from the Nanjing Biomedical Research Institute of Nanjing University (Jiangsu, China). The mice were housed in plastic cages in which the humidity and temperature were controlled at 50–60% and 20 ± 2°C. Fenofibrate was dissolved in 0.5% sodium carboxymethyl cellulose (CMC-Na). Mice in the control group were fed a normal diet, while mice in the other group were fed HFD (HFD-fed group), which consisted of 20% carbohydrate, 20% protein, and 60% fat (total 25.07 kJ/g), for 14 weeks. The HFD-fed mice were administered CMC-Na (0.5%) daily via oral gavage for the last 4 weeks of treatment. For the fenofibrate + HFD-fed group, HFD-fed mice were administered fenofibrate (40 mg/kg) via oral gavage daily for the last 4 weeks of treatment. Body weight was measured once a week throughout the study. The animal experiment complied with the ARRIVE guidelines and the National Institutes of Health Guide for the Care and Use of Laboratory Animals. All protocols were approved by the Institutional Animal Care and Use Committee of Kunming Medical University (Approval no. kmmu2020248).

### Western Blotting

Mouse livers were lysed in lysis buffer on ice for 1 h. Protein lysates were loaded into each well (20 μg/10 μl) and separated on SDS-PAGE (7.5, 10, or 12.5%). Proteins were transferred onto PVDF membranes (Millipore, Bedford, MA, United States) and blocked with 5% skim milk-TBST for 2 h at 20°C. The primary antibodies used were anti-ATP2A2/SERCA2b (#4388, Cell Signaling Technology, Beverly, MA, United States), anti-BiP (#3183, Cell Signaling Technology), anti-CCAAT/enhancer-binding homologous protein (CHOP) (#2895, Cell Signaling Technology), and anti-GAPDH (#8884, Cell Signaling Technology). Immunoreactive signals were detected using ECL substrate reagents (#32109, Thermo Scientific Science, Waltham, MA, United States), followed by image analysis (Amersham Imager 600). Quantification was performed using ImageJ (NIH).

### Quantitative Real-Time PCR

Total miRNA in tissue was extracted with the miRcute miRNA Isolation kit (DP501, Transgen, Being, China). cDNA was generated using the miRcute Plus miRNA First-Strand cDNA kit (KR211-01, Trangen, Being, China). qRT-PCR was performed using the miRcute Plus miRNA qPCR kit (FP411-01-01, Trangen) on a Roche LightCycler 480 System. The primers used for the qRT-PCR assay are listed in Supplementary Table S1. All qRT-PCR analyses were performed in triplicate. For each gene, mRNA expression was normalized to that of *U6*, and the fold change in mRNA expression was determined using the 2^−ΔΔCT^ method.

### ELISA

After sevoflurane anesthesia, blood was collected from the heart, and fasting blood glucose level was measured. The supernatant was collected, and the serum insulin content was detected using a Mouse INS (Insulin) ELISA Kit (E-EL-M1382c, Elabscience, Wuhan, China). The insulin resistance (IR) and sensitivity indices were calculated using the following formulae: IR index = [fasting blood glucose (mmol/L) × serum insulin (mIU/L)]/22.5 and insulin sensitivity index = 1/[fasting blood glucose (mmol/L) × serum insulin (mIU/L)] (Fang et al., 2010). The supernatant of liver tissue homogenate and serum were used to measure TG content using a TG assay Kit (E-BC-K261, Elabscience).

### Target Sites Analysis

We use the public prediction platform TargetScan (http://www.targetscan.org/) and MicroRNA Target Prediction Database (miRDB, URL: http://mirdb.org/) to search potential 3′ UTR of *SERCA2b* that let-7 family members bind.

### Luciferase Reporter Assay

The 3′ UTR of *SERCA2b* mRNA was amplified and cloned into the psiCHECK2 vector. HepG2 human hepatocarcinoma cells were seeded onto 24-well plates and cultured in DMEM (Hyclone, Logan, UT) supplemented with 10% fetal bovine serum (Hyclone) at 37°C and 5% CO_2_. At 60–80% confluence, the cells were transfected using Lipofectamine 2000® Reagent (Thermo Fisher Scientific) with the 3′ UTR of SERCA2b reporter plasmids with miR-let-7 mimic (5′-AUG​UUG​GAU​GAU​GGA​GUC​UUC-3′) or negative control (NC) mimic (5′-CAG​UAC​UUU​UGU​GUA​GUA​CAA-3′). At 48 h after transfection, the Dual Luciferase Assay System (Promega, Beijing, China) was used to determine luciferase activity. Renilla luciferase activity was used for normalization in each well.

### Histology and Immunohistochemistry

Fresh liver tissues were fixed in 10% formalin, embedded in paraffin, and sectioned at a thickness of 5 μm. To measure hepatic macrosteatosis, the sections were stained with hematoxylin and eosin. To determine fat deposition in each group, the sections were stained with Oil Red O staining solution for 10 min at 60°C. The paraffin-embedded liver tissues were also sliced into 4-μm-thick sections and stained with Masson’s trichrome to observe the degree of liver tissue fibrosis. For immunohistochemical analysis, the sections were stained with anti-ATP2A2/SERCA2b (1:800, #4388, Cell Signaling Technology) or anti-F4/80 antibody (Proteintech, Wuhan, China) per manufacturer’s instructions. Images were acquired using a Leica Aperio CS2 system. Immunohistochemical staining was used to determine the expression of SERCA2b at the protein level, and the expression was quantified using the Image-Pro Plus 6.0 software. The stained sections were assessed at ×400 magnification, and five representative staining fields of view in each section were analyzed. The total area of staining was divided by the total area of the slide (mm^2^) and multiplied by 100 to obtain the percentage of the stained area.

### Statistical Analyses

Data are presented as the mean ± SD. Normal distribution was checked using Shapiro-Wilk test. Statistical differences between two groups were analyzed using Student’s *t*-test. Statistical analyses between multiple groups were performed using one-way analysis of variance with Student-Newman-Keuls test. GraphPad Prism 7 was used to analyze the data. *p* < 0.05 was considered statistically significant.

## Results

### Fenofibrate Improves Hepatic Steatosis and Insulin Resistance in High-Fat Diet-Fed Mice

**FIGURE 1:**
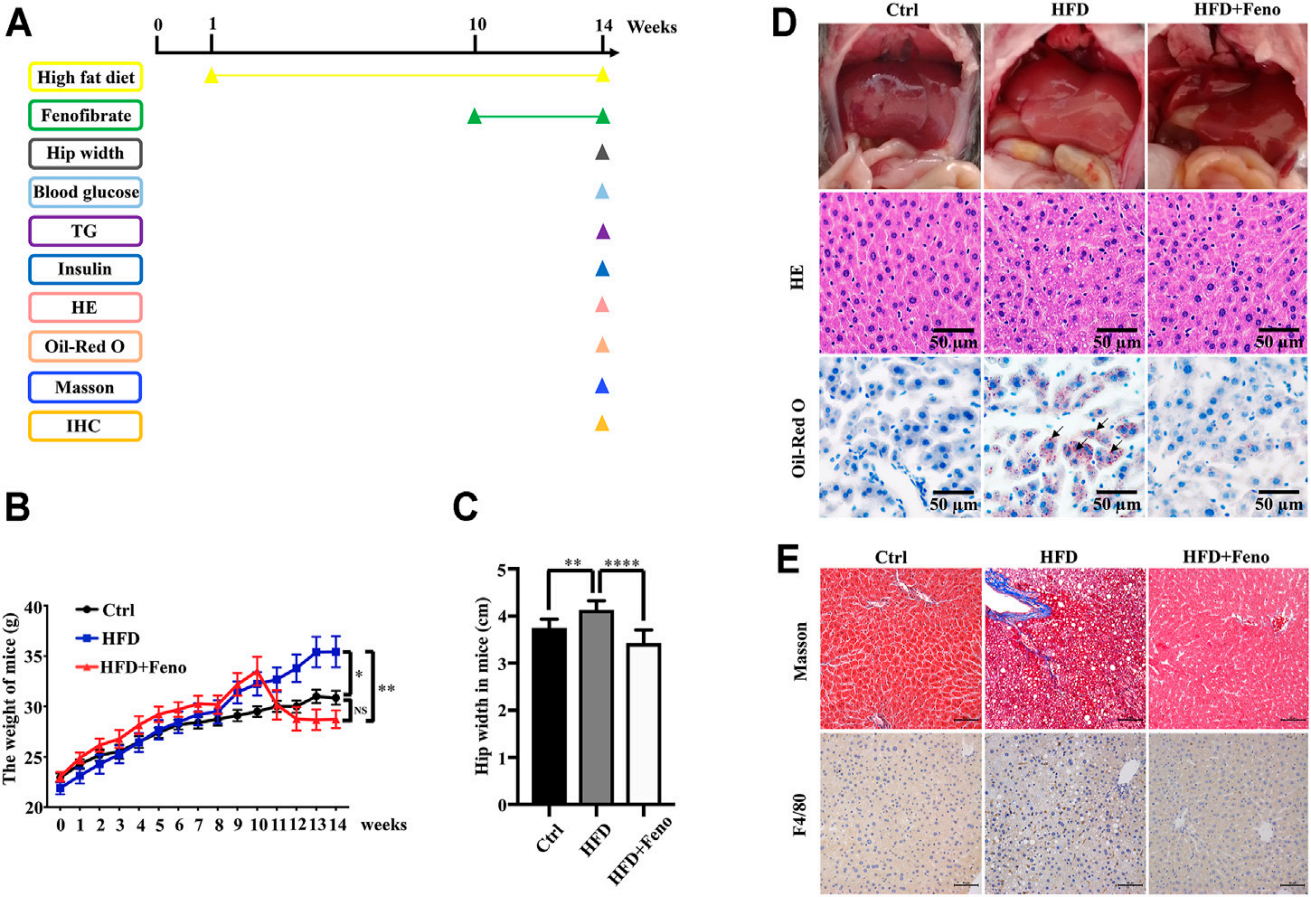
Fenofibrate attenuates body mass, liver histology in HFD-fed mice. **(A)** Mice experiment process plan diagram. Effects of fenofibrate on bodyweight **(B)** and hip-width **(C)** after 14 weeks. These results are means ± SD (*n*=6−10 mice/group). *p < 0.05, **p < 0.01, ****p < 0.0001, HFD group versus control group, or fenofibrate + HFD-fed group versus HFD group. **(D)** Liver gross morphology, hepatic macrosteatosis, and fat deposition of mice in each group (original magnification ×200; scale bar, 50 μm). **(E)** Masson staining and F4/80 staining of liver tissue in each group (magnification ×200; scale bar, 50 μm).

**FIGURE 2:**
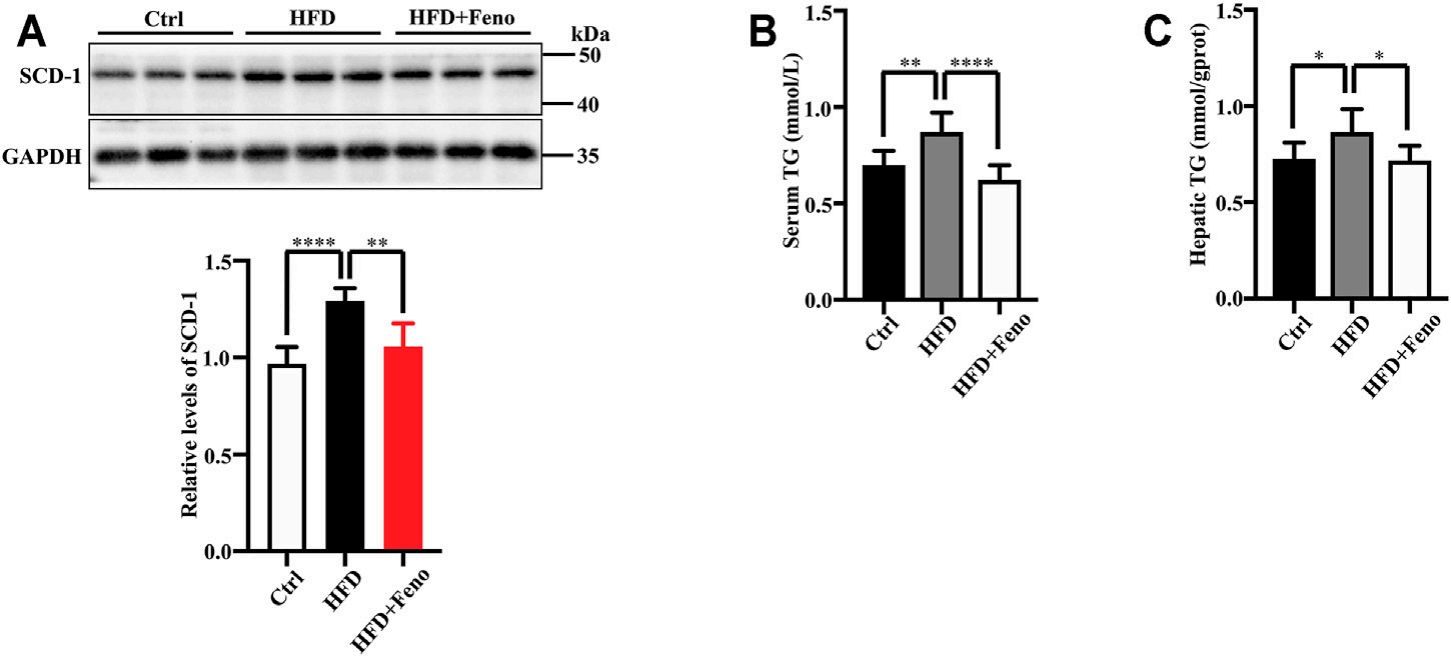
Fenofibrate attenuates lipid accumulation in HFD-fed mice. **(A)** The levels of SCD-1 were measured by western blotting after 14 weeks. Representative western blots are shown. These results are means ± SD (*n* = 5–8 mice/group). **p < 0.01, ****p < 0.0001, HFD group versus control group, or fenofibrate + HFD-fed group versus HFD group. **(B)** Serum triglyceride (TG) levels and **(C)** hepatic TG levels. These results are means ± SD (*n* = 10). *p < 0.05, **p < 0.01, ****p < 0.0001, HFD group versus control group, or fenofibrate + HFD-fed group versus HFD group.

**FIGURE 3:**
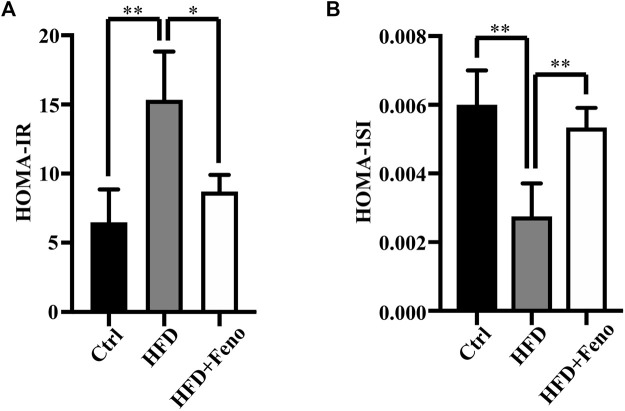
Effects of fenofibrate on insulin resistance in HFD-fed mice. **(A)** HOMA- IR and **(B)** HOMA-ISI. These results are means ± SD (*n*=3−4 mice/group). *p < 0.05, **p < 0.01, HFD group versus control group, or fenofibrate + HFD-fed group versus HFD group.

After 14 weeks of HFD feeding, the mice became obese, and this was accompanied by an increase in body weight and hip width (Figures 1B,C). To confirm the beneficial effects of fenofibrate, these DIO mice were administered vehicle (CMC-Na) and fenofibrate via oral gavage daily for 4 weeks (Figure 1A). The body weight and average hip width of DIO mice significantly decreased after fenofibrate administration. Compared with mice administered the vehicle, DIO mice treated with fenofibrate exhibited a significant decrease in hepatocyte ballooning (Figure 1D), macrosteatosis, and fat deposition in the liver (Figure 1D) as well as the levels of serum and hepatic TG (Figures 2B,C). We used Masson staining to detect the fibrosis of liver, and a reduction in liver fibrosis was observed after fenofibrate treatment. Additionally, we measured the expression of F4/80, a macrophage-specific marker; the proportion of F4/80-positive cells in mice in the fenofibrate + HFD-fed group was significantly decreased compared with the HFD-fed group (Figure 1E). Moreover, the expression of stearoyl-CoA desaturase (SCD-1), a key enzyme for monounsaturated fatty acid synthesis (Poloni et al., 2015), was markedly upregulated in DIO mice, and it was reduced by fenofibrate treatment (Figure 2A). Furthermore, IR is one of the most frequent complications of obesity; thus, the effects of fenofibrate on IR were determined. The results indicated that fenofibrate treatment reduced homeostasis model assessment-insulin resistance (HOMA-IR) values (Figure 3A; Supplementary Table S2) and increased homeostasis model assessment-insulin sensitivity index (HOMA-ISI) (Figure 3B; Supplementary Table S2). These data indicate that fenofibrate treatment markedly attenuates NAFLD phenotypes in DIO mice.

### Fenofibrate Treatment Ameliorates Endoplasmic Reticulum Stress Accompanied by the Upregulation of SERCA2b Expression in the Liver of High-Fat Diet-Fed Mice

**FIGURE 4:**
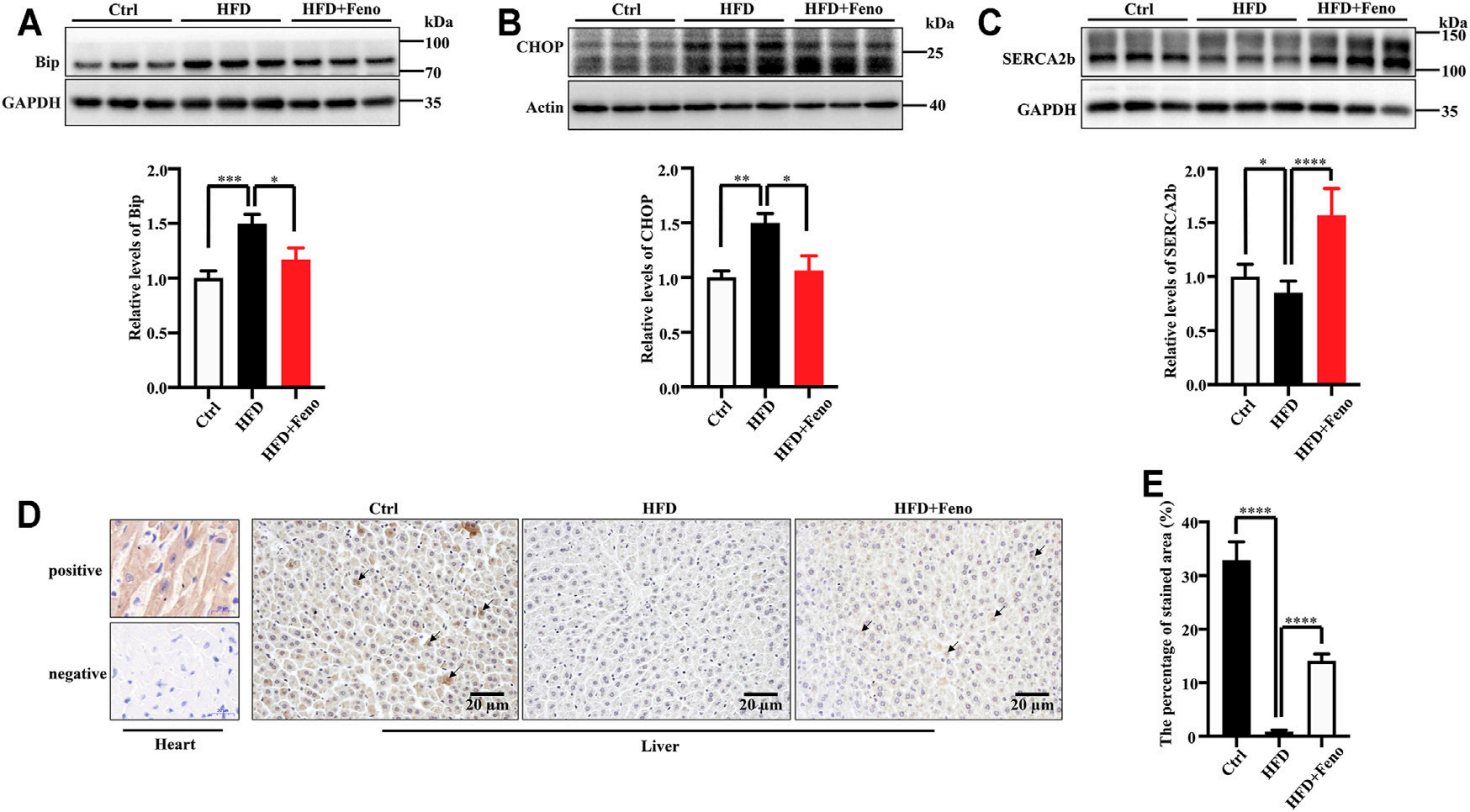
Fenofibrate alleviates ER stress and elevates the expression of SERCA2b. The expression of Bip **(A)**, CHOP **(B)**, and SERCA2b **(C)** were measured by western blotting. Representative western blots are shown. These results are means ± SD (*n* = 5–9 mice/group). *p < 0.05, **p < 0.01, ***p < 0.001, ****p < 0.0001, HFD group versus control group, or fenofibrate + HFD-fed group versus HFD group. **(D)** SERCA2b expression was determined by immunohistochemistry (scale bar, 20 μm); heart tissue was used as a positive and a negative control. **(E)** The percentage of stained area in immunohistochemistry. ****p < 0.0001, HFD group versus control group, or fenofibrate + HFD-fed group versus HFD group.

ER stress plays a crucial role in the pathology of hepatic steatosis (Puri et al., 2008). Therefore, we tested whether ER stress, activated by HFD feeding, was attenuated by fenofibrate treatment. The expression of two indicators of ER stress at the protein level, BiP and CHOP, was upregulated in DIO mice (Figures 4A,B) compared with mice fed a normal diet. Fenofibrate treatment markedly ameliorated ER stress, which was activated by HFD feeding (Figures 4A,B). The expression or activity of SERCA2b is significantly reduced (Park et al., 2010; Fu et al., 2011; Dai et al., 2019), while increased SERCA2b can ameliorate ER stress in obese mice (Kang et al., 2016; Ouyang et al., 2017). To identify whether the amelioration of ER stress is associated with SERCA2b, the protein levels of SERCA2b were measured by western blotting. Indeed, fenofibrate treatment significantly restored the protein levels of SERCA2b in DIO mice (Figure 4C). Similar results were confirmed by immunohistochemical analysis (Figures 4D,E). These results indicate that fenofibrate treatment ameliorates ER stress and simultaneously upregulates the expression of SERCA2b in the liver of HFD-fed mice.

### Fenofibrate Treatment Attenuates Let-7 Expression in High-Fat Diet-Fed Mice

**FIGURE 5:**
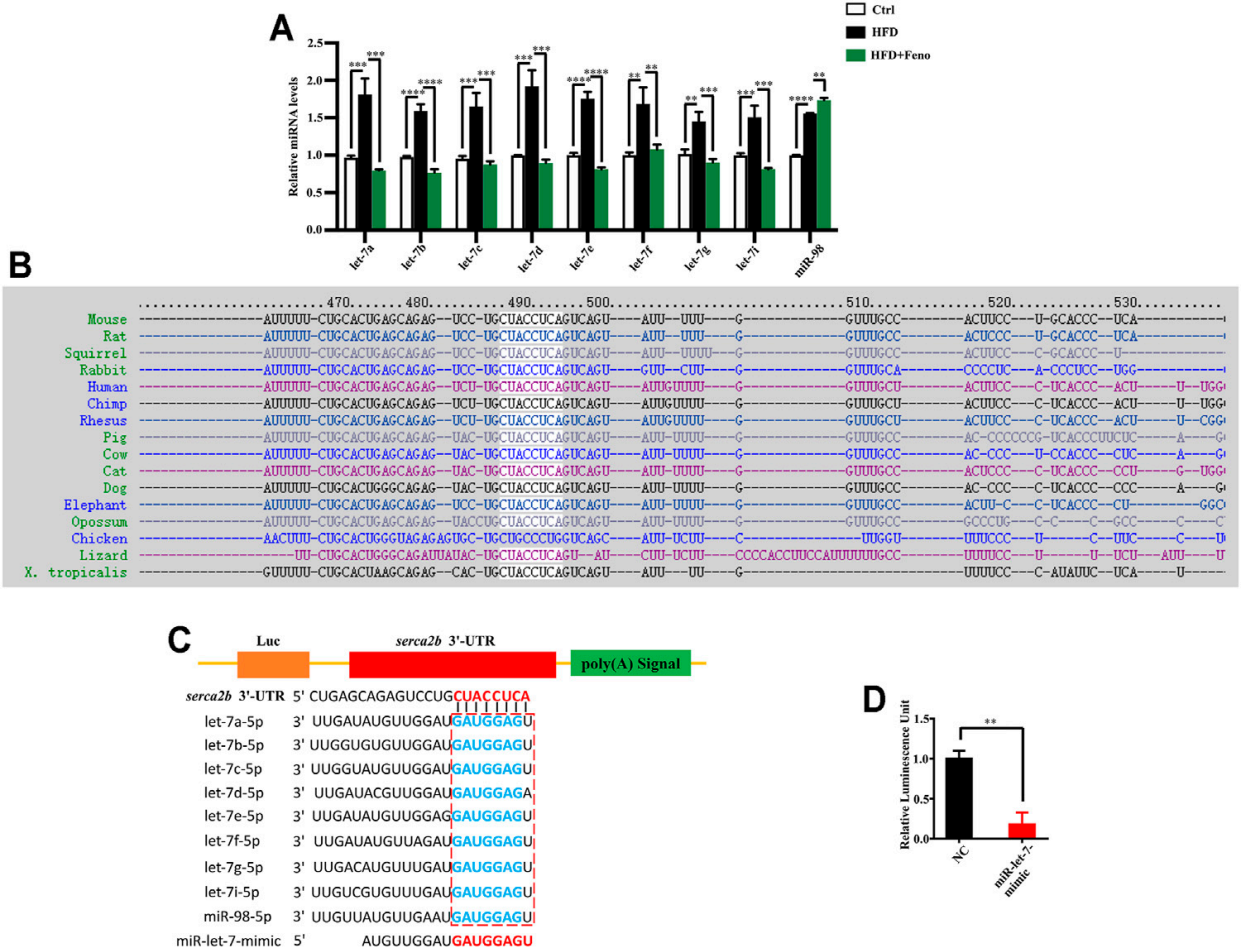
Let-7 regulates the expression of SERCA2b. **(A)** qPCR analysis confirmed that fenofibrate attenuates let-7 expression in HFD-fed mice after 14 weeks. These results are means ± SD (*n*=3−9 mice/group). **p < 0.01, ***p < 0.001, ****p < 0.0001, HFD group versus control group, or fenofibrate + HFD-fed group versus HFD group. **(B)** Sequences of the *SERCA2b* in different species. *SERCA2b* is highly conserved in different species. **(C)** Complementarity between the 3′ UTR of *SERCA2b* mRNA and let-7. The box indicates the seed region of the let-7 family. **(D)** Luciferase analysis of a reporter vector harboring the 3′ UTR of *SERCA2b* in HepG2 cells transfected with a negative control (NC) mimic or miR-let-7 mimic together with psiCHECK2-*SERCA2b* 3′ UTR reporter plasmids for 48 h. These results are means ± SD of three independent experiments. **p < 0.01, miR-let-7 mimic group versus negative control group.

Overexpression of let-7 disrupts glucose tolerance, while global knockdown of let-7 improves impaired glucose tolerance in DIO mice (Frost and Olson, 2011). Using miRNA microarrays, our recent study indicated that let-7 family miRNA levels are elevated in HFD-fed rats (Dai et al., 2019). There are 12 members in the let-7 family in mice (Papaioannou et al., 2013). Therefore, to investigate whether fenofibrate regulates the expression of let-7 family members, we measured the levels of nine let-7 family members (let-7a, let-7b, let-7c, let-7d, let-7e, let-7f, let-7g, let-7i, and miR-98) in the liver of mice using qRT-PCR and found that the levels of let-7a, let-7b, let-7c, let-7d, let-7e, let-7f, let-7g, let-7i, and miR-98 were increased in DIO mice (Figure 5A). These results indicate that fenofibrate treatment significantly inhibits the levels of let-7 family members in the liver of DIO mice.

### *SERCA2b* is a Target Gene of Let-7

*SERCA2b* sequence is highly conserved in different species, including humans and rodents (Figure 5B). To identify whether SERCA2b is regulated by let-7, the sequence alignment of *SERCA2b* 3′ UTR and let-7 family members was analyzed using the TargetScan algorithm. The data indicated that let-7 family members could bind to the 3′ UTR of *SERCA2b* (Figure 5C; Supplementary Table S3). To confirm the involvement of let-7 in the regulation of SERCA2b expression, the 3′ UTR of *SERCA2b* was cloned into a reporter vector, and transfection with miR-let-7 mimics markedly decreased the luciferase activity of the 3′ UTR of *SERCA2b* in HepG2 cells (Figure 5D). These data demonstrate that let-7 miRNA directly targets *SERCA2b*.

## Discussion

**FIGURE 6:**
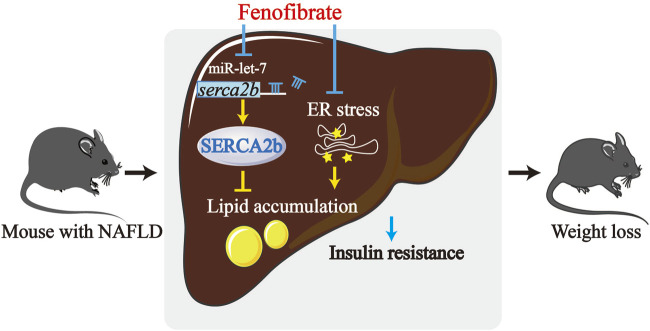
The mechanism of fenofibrate that leads to improved hepatic lipid accumulation and insulin resistance. The levels of let-7 are upregulated in HFD-induced obese (DIO) mice. Fenofibrate reduced the levels of the let-7 family members and upregulated the levels of SERCA2b, which is a target of let-7. Fenofibrate treatment also attenuated insulin resistance and suppressed excessive lipid accumulation in HFD-fed mice. Thus, the protective effect of fenofibrate could be reasonably related to alteration of the let-7/SERCA2b axis and alleviation of ER stress.

In this study, the possible molecular mechanism underlying the fenofibrate-mediated alleviation of IR and hepatic steatosis in a rodent model of NAFLD was investigated (Figure 6). The expression of let-7 family members was upregulated in DIO mice, which were effectively reduced by fenofibrate. Further investigation revealed that let-7 regulated the expression of *SERCA2b* by directly targeting the 3′ UTR of *SERCA2b*. Additionally, fenofibrate elevated the levels of SERCA2b. Thus, the effect of fenofibrate might be associated with the let-7/SERCA2b signaling pathway in the liver of HFD-induced NAFLD mice.

Increasing evidences demonstrated that activation of ER stress initiates hepatic steatosis, inflammation, and IR, all of which are vital factors involved in the pathogenesis of NAFLD, inhibition of ER stress is recognized as a potential therapeutic strategy for NAFLD. As a PPAR-α agonist, fenofibrate has been widely used in the treatment of dyslipidemia (Liu et al., 2014), it can effectively against NAFLD and improve liver function (Prisingkorn et al., 2017), and the probable mechanism is that fenofibrate activates PPARα, which can prevent excessive hepatic triglyceride (TG) accumulation (Kostapanos et al., 2013). While, recent studies have shown that fenofibrate can alleviate ER stress in mice (Zhang et al., 2015), but the specific mechanism remaines largely unclear. In the current study, we found that fenofibrate attenuated the expression of BiP and CHOP, which are the indicators of ER stress, and their expression is upregulated in NAFLD. Moreover, fenofibrate increased the levels of SERCA2b, which is a regulator of ER stress. Protein and mRNA levels of SERCA2b are reduced in the liver of obese mice, while, overexpression of SERCA2b dramatically alleviates ER stress, increases insulin sensitivity in obese mice (Park et al., 2010). SERCA2b overexpression significantly suppresses steatosis by inhibiting the upregulation of the expression of lipogenic genes (*DGAT2*, *SCD1*, and *ACC2*) in ob/ob mice (Park et al., 2010); however, SERCA2b overexpression does not influence the expression of SREBP1c, a master regulator of the aforementioned lipogenic genes. Thus, downregulation of the expression of these lipogenic genes by SERCA2b is independent of SREBP1c. Moreover, the levels of SCD1, DGAT2, and ACC2 have been found to be directly regulated by XBP1 in the liver (Lee et al., 2008). Thus, SERCA2b probably regulates lipid accumulation via XBP1-mediated inhibition of lipogenic genes. In addition to XBP1, some other transcriptional factors may also regulate the expression of SERCA2b, such as pancreatic and duodenal homeobox protein 1, which regulates SERCA2b expression in β-cells to maintain ER calcium levels (Johnson et al., 2014).

Dysregulation of miRNA expression has been associated with NAFLD through the alteration of various pathways (Trajkovski et al., 2011; Ng et al., 2014; Wu et al., 2017; Dai et al., 2019). For example, using miRNA microarrays, our recent study identified that the upregulation of miR-30b expression promotes hepatic steatosis and IR by targeting SERCA2b in HFD-fed rats (Dai et al., 2019). Let-7 family has emerged as a central regulator of energy which involved in the regulation of glucose metabolism (Jiang, 2019), its expression is elevated in HFD-fed rats (Dai et al., 2019). Overexpression of let-7 g results in impaired glucose tolerance in mice (Frost and Olson, 2011), knockdown of let-7 family could be sufficient to attenuate glucose tolerance and insulin resistance in obese mice (Frost and Olson, 2011). Previous studies have demonstrated that let-7 induces hepatic lipid accumulation and IR by targeting the key components of the insulin signaling pathway, such as IRS2, INSR, and IGF1R, in obese mice (Frost and Olson, 2011). In this study, the levels of let-7 family members were also assessed in DIO mice. Fenofibrate treatment downregulated the expression of let-7 family members in DIO mice, furthermore, the data presented in this study demonstrate that *SERCA2b* is a novel target of let-7. Thus, fenofibrate appears to regulate the let-7/SERCA2b signaling pathway in NAFLD mice.

In conclusion, the data demonstrate that fenofibrate alleviates ER stress and upregulates the expression of SERCA2b, a regulator of ER stress, in NAFLD mice. Moreover, the expression of let-7 is regulated by fenofibrate, while let-7 regulates SERCA2b by directly targeting the 3′ UTR of *SERCA2b*. Collectively, our findings suggest that the protective effects of fenofibrate in NAFLD mice could be related to the alleviation of ER stress and regulation of the let-7/SERCA2b axis. Therefore, an improved understanding of the mechanism of action of fenofibrate in NAFLD could lead to the development of highly effective treatments for lipid metabolism disorders or ER stress-associated diseases. Furthermore, as the safety and specific mechanisms of action of fenofibrate are well established, it can become more widely used in clinical therapy.

## Data Availability

The original contributions presented in the study are included in the article/Supplementary Material, further inquiries can be directed to the corresponding authors.

## Abbreviations

ATF6, activating transcription factor 6; CHOP, CCAAT/enhancer-binding homologous protein; CMC-Na, sodium carboxymethyl cellulose; DIO, diet-induced obese; ER, endoplasmic reticulum; HFD, high-fat diet; HOMA-IR, homeostasis model assessment-insulin resistance; HOMA-ISI, homeostasis model assessment-insulin sensitivity index; INS, Insulin; IR, insulin resistance; IRE1α, inositol-requiring enzyme 1α; miRNAs, MicroRNAs; NAFLD, non-alcoholic fatty liver disease; NC, negative control; Pdx-1, pancreatic and duodenal homeobox protein 1; PPAR-α, peroxisome proliferator-activated receptor α; SCD-1, stearoyl-CoA desaturase; SERCA, sarco/endoplasmic reticulum calcium ATPase; TG, triglyceride; UPR, Unfolded protein response; UTR, untranslated region, XBP1; X-box binding protein 1.
