# Supplementary material for: Fenofibrate Improves Insulin Resistance and Hepatic Steatosis and Regulates the Let-7/SERCA2b Axis in High-Fat Diet-Induced Non-Alcoholic Fatty Liver Disease Mice
Source: Front Pharmacol. 2022 Jan 19;12:770652. doi: 10.3389/fphar.2021.770652 (PMC8807641; doi:10.3389/fphar.2021.770652)
Supplement: Supplementary file 1 [file Table1.DOCX]

**Supplementary Table 1.** The primers used for qRT-PCR assay.

| Gene | Species | sequence |
| --- | --- | --- |
| let-7a  let-7b  let-7c  let-7d  let-7e  let-7f  let-7g  let-7i  mir-98 | mouse  mouse  mouse  mouse  mouse  mouse  mouse  mouse  mouse | 5′- UGAGGUAGUAGGUUGUAUAGUU- 3′  5′- UGAGGUAGUAGGUUGUGUGGUU- 3′  5′- UGAGGUAGUAGGUUGUAUGGUU- 3′  5′- AGAGGUAGUAGGUUGCAUAGUU- 3′  5′- UGAGGUAGGAGGUUGUAUAGUU- 3′  5′- UGAGGUAGUAGAUUGUAUAGUU- 3′  5′- UGAGGUAGUAGUUUGUACAGUU- 3′  5′- UGAGGUAGUAGUUUGUGCUGUU- 3′  5′- UGAGGUAGUAAGUUGUAUUGUU- 3′ |
