# Supplementary material for: Fenofibrate Improves Insulin Resistance and Hepatic Steatosis and Regulates the Let-7/SERCA2b Axis in High-Fat Diet-Induced Non-Alcoholic Fatty Liver Disease Mice
Source: Front Pharmacol. 2022 Jan 19;12:770652. doi: 10.3389/fphar.2021.770652 (PMC8807641; doi:10.3389/fphar.2021.770652)
Supplement: Supplementary file 2 [file Table2.docx]

**Supplementary Table 2.** The levels of fasting blood-glucose and insulin to calculate the HOMA-IR and HOMA-ISI.

| Group | Fasting blood-glucose（mmol/L） | Insulin（mIU/L） | HOMA-IR | HOMA-ISI |
| --- | --- | --- | --- | --- |
| Ctrl | 4.375 ± 0.695 | 34.490 ± 15.440 | 6.481 ± 2.385 | 0.006 ± 0.001 |
| HFD | 6.400 ± 0.625 | 53.720 ± 8.849 | 15.350 ± 3.477** | 0.003 ± 0.001** |
| HFD+Feno | 5.200 ± 1.833 | 40.160 ± 11.830 | 8.709 ± 1.196**^#^** | 0.005 ± 0.001**^##^** |
| *F* | 2.751 | 1.968 | 11.11 | 13.62 |
| *P* | 0.131 | 0.210 | 0.007 | 0.004 |

Data presents mean ± SD (*n* = 3-4 mice/group). ***P* < 0.01, HFD group versus control group, **^#^***P* < 0.05, **^##^***P* < 0.01, fenofibrate+HFD-fed group versus HFD group, respectively, using one-way ANOVA.
