# Supplementary material for: Fenofibrate Improves Insulin Resistance and Hepatic Steatosis and Regulates the Let-7/SERCA2b Axis in High-Fat Diet-Induced Non-Alcoholic Fatty Liver Disease Mice
Source: Front Pharmacol. 2022 Jan 19;12:770652. doi: 10.3389/fphar.2021.770652 (PMC8807641; doi:10.3389/fphar.2021.770652)
Supplement: Supplementary file 3 [file Table3.DOCX]

**Supplementary Table 3.** Predicted targeting details, *SERCA2b* is a target of let-7 family.

| **miRNA** | **Position in the UTR** | **seed match** | **context++ score** | **context++ score percentile** | **weighted context++ score** | **conserved branch length** | **Pct** |
| --- | --- | --- | --- | --- | --- | --- | --- |
| **let-7a-5p** | 4391-4397 | 7mer-1A | -0.18 | 72 | -0.16 | 3.726 | 0.91 |
| **let-7b-5p** | 4391-4397 | 7mer-1A | -0.18 | 72 | -0.16 | 3.726 | 0.91 |
| **let-7c-5p** | 4391-4397 | 7mer-1A | -0.18 | 72 | -0.16 | 3.726 | 0.91 |
| **let-7d-5p** | 4391-4397 | 7mer-1A | -0.21 | 74 | -0.18 | 3.726 | 0.91 |
| **let-7e-5p** | 4391-4397 | 7mer-1A | -0.18 | 72 | -0.16 | 3.726 | 0.91 |
| **let-7f-5p** | 4391-4397 | 7mer-1A | -0.17 | 69 | -0.15 | 3.726 | 0.91 |
| **let-7g-5p** | 4391-4397 | 7mer-1A | -0.21 | 76 | -0.18 | 3.726 | 0.91 |
| **let-7i-5p** | 4391-4397 | 7mer-1A | -0.21 | 76 | -0.18 | 3.726 | 0.91 |
| **miR-98-5p** | 4391-4397 | 7mer-1A | -0.19 | 74 | -0.17 | 3.726 | 0.91 |
